# Sugar-sweetened beverage tax implementation processes: results of a scoping review

**DOI:** 10.1186/s12961-022-00832-3

**Published:** 2022-03-24

**Authors:** Sarah Forberger, Lucia Reisch, Biljana Meshkovska, Karolina Lobczowska, Daniel Alexander Scheller, Janine Wendt, Lara Christianson, Jennifer Frense, Jürgen Michael Steinacker, Aleksandra Luszczynska, Hajo Zeeb, on behalf of the PEN Consortium

**Affiliations:** 1 Department of Prevention and Evaluation, Leibniz Institute for Prevention Research and Epidemiology-BIPS, Achterstraße 30, 28359 Bremen, Germany; 2 El-Erian Professor of Behavioural Economics and Policy, University of Cambridge, Cambridge, CB3 9ET United Kingdom; 3 Institute of Basic Medical Sciences, University of Oslo, Domus Medica, Gaustad, Sognsvannsveien 9, 2. etg., 0372 Oslo, Norway; 4 Department of Psychology in Wroclaw, SWPS University of Social Sciences and Humanities, Ostrowskiego Street 30b, 53238 Wroclaw, Poland; 5 Division of Sports and Rehabilitation Medicine, Department of Internal Medicine II, Ulm University Medical Center, Leimgrubenweg 14, 89075 Ulm, Germany; 6 National Institute for Human Resilience, University of Colorado at Colorado Springs, 1420 Austin Bluffs Pkwy, CO Springs, CO 80918 United States of America; 7 Health Sciences Bremen, University of Bremen, Bibliothekstr.1, 28359 Bremen, Germany

**Keywords:** SSB taxation, Public policy, Implementation, Implementation process

## Abstract

Taxing sugar-sweetened beverages (SSB) is seen as a win–win situation for governments. It is argued that SSB taxes are relatively easy to implement from a practical perspective compared to for example other nutrition policies. However, the implementation of SSB taxation laws does not happen by itself. Therefore, this work examines implementation processes for SSB taxation in terms of (1) pre-implementation context, (2) taxation instruments used and (3) interactions in the implementation process. Ten databases and grey literature were systematically searched for studies reporting on SSB taxation implementation processes up to February 2020. All studies (*N* = 1248) were screened independently by two reviewers according to predefined criteria. The selection of variables to be extracted was based on the policy cycle heuristic and informed by intervention implementation research. Information on the process of implementing SSB taxation is limited. Only six cases based on three publications were identified, indicating a gap in this research area. SSB taxation implementation was accomplished by hiring a subcontractor for the implementation or using pre-existing tax collection structures. Political and public support within the implementation process seems to be supportive for the city of Berkeley and for Portugal but was not reported for the Pacific Islands. However, the existing data are very limited, and further research on SSB taxation implementation processes is needed to determine whether the aim of the policy and the envisaged outcome are linked in practice.

*Registration* The protocol was registered with the Open Science Framework (OSF) (osf.io/7w84q/)

**Supplementary Information:**

The online version contains supplementary material available at 10.1186/s12961-022-00832-3.

## Background

The consumption of sugar-sweetened beverages (SSB) is positively associated with a number of health risks (Table 1). Further, obesity rates in men and women have increased worldwide and are still on the rise [1, 2]. Therefore, reducing high-calorie beverage consumption may help to reduce obesity [3–5].

**Table 1:** Summary of evidence of health risks linked to SSB consumption (table adapted from World Bank 2020 [1]

Health risks	Nature of evidence	Key references
Weight gain, overweight, obesity	Strong, consistent evidence of direct, causal relationship	Bleich and Vercammen 2018 [6]; Malik et al. 2013 [7]; Te Morenga et al. 2012 [8]; Trumbo and Rivers 2014 [9]
Type 2 diabetes	Strong positive association (independent and BMI-mediated)	Imamura 2015 [10]; Malik 2010 [11]; Schulze et al. 2004 [12]
Dental caries	Strong positive dose–response relationship	Bleich and Vercammen 2018 [6]
Metabolic syndrome	Positive association (independent and BMI-mediated)	Malik et al. 2010 [11]
CVD risk factors and outcomes	Strong positive association with CHD (independent and BMI-mediated); association with stroke less clear	Fung et al. 2009 [13]; de Koning et al. 2012 [14]; Malik et al. 2010 [15]; Malik and Hu 2019 [16]; Te Morenga et al. 2014 [17]; Xi 2015 [18]
Cancer	Positively associated with increased risk of at least 12 cancers (independent and BMI-mediated)	Chazelas et al. 2019 [19]; Guh et al. 2009 [20]; Mueller et al. 2010 [21]; WCRF and AICR 2018 [22]
All-cause and cause-specific mortality	Positively associated with higher risk of death from all causes. Linked to 184,000 deaths worldwide: 76% in low- and middle-income countries and 72% related to type 2 diabetes	Mullee et al. 2019 [23]; Singh et al. 2015 [24]

*BMI* body mass index, *CHD* coronary heart disease, *CVD* cardiovascular disease

**Fig. 1:**
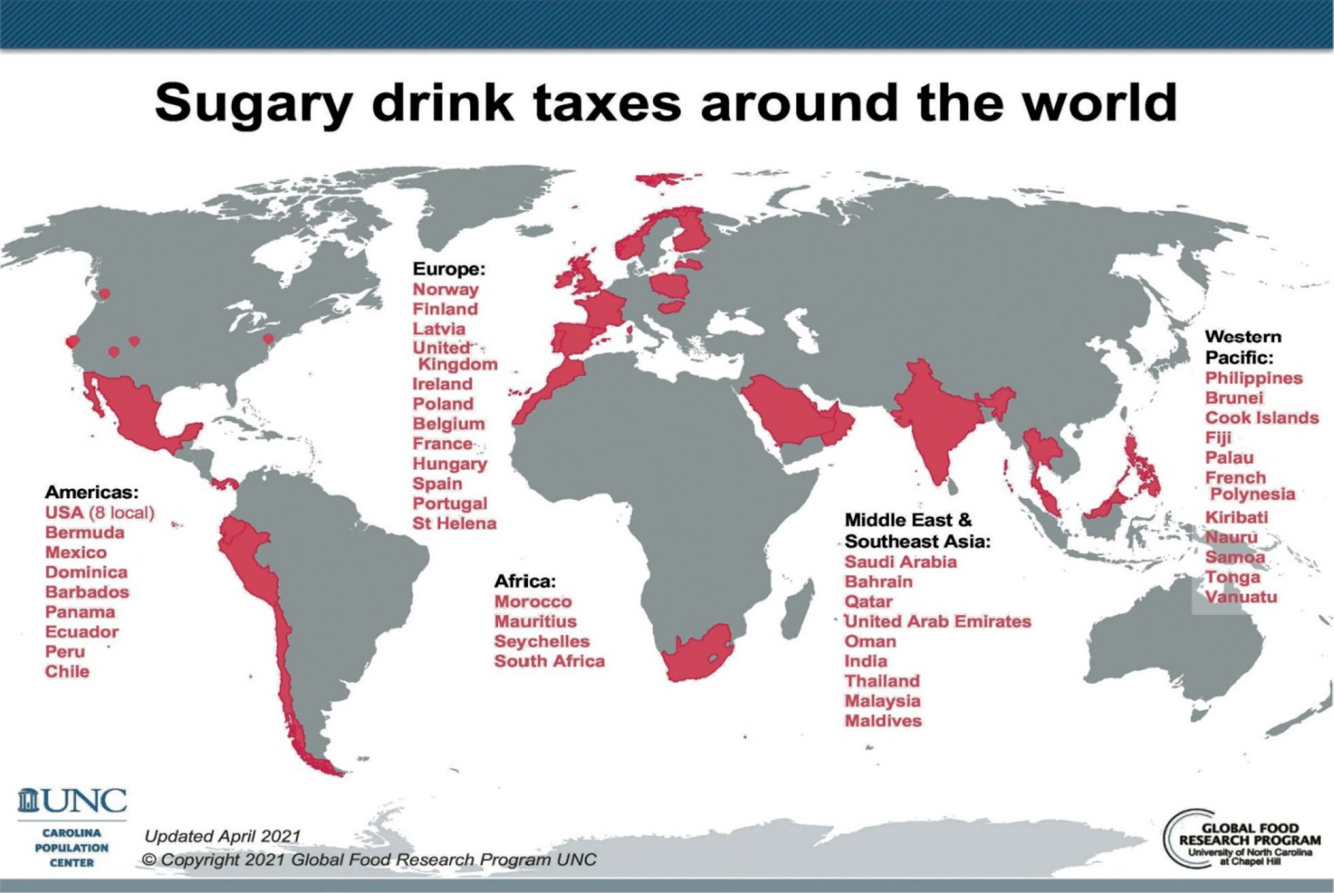
Worldwide SSB taxes (April 2021; ©2021 Global Food Research Program, University of North Carolina at Chapel Hill (UNC); permission obtained)

SSB taxation is seen as a win–win situation for governments. The tax can trigger shifts in consumption and purchasing behaviour, incentivize product reformulation, and increase government revenues to fund public services and goods [6–17]. Currently, over 45 countries worldwide have implemented a tax on SSBs (Additional file 1: Appendix 1 Table A.1, Fig. 1; [18–20]).

However, little is known about the actual implementation process, namely what happens after the tax legislation is passed [21, 22].

The term SSB taxation encompasses a variety of objectives and mechanisms that condition the feasibility and acceptability of the tax and modify its impact [15]. SSB taxes differ in terms of tax objectives (changing consumption, generating revenue, incentivizing reformulation), type of tax, tax rate and scope (e.g. including or excluding zero-calorie sweetened beverages, candy, ice cream), tax jurisdiction (local, subnational, national) and use of tax revenue (Additional file 1: Annex 1 Table A1.1 and A1.2; [15, 23, 24]. Understanding how the tax is implemented is valuable for overcoming implementation barriers that could lead to delays in revenue, distortions in dealing with businesses, and decreasing citizen acceptability [15, 25]. Further aligning implementation with tax system capacities increases the likelihood of compliance and enforcement, and thus their effectiveness. Tax capacity is the ability of a state to implement and monitor taxation, build effective structures, train staff, and offer effective fiscal services and monitoring systems for tax transaction [26, 27]. In practice, there can be a wide gap between the tax in law and the tax in the treasury [28, 29].

Following policy-making as a process often labelled as policy cycle, policy-making is basically divided into agenda-setting, formulation, adoption, implementation, evaluation and support/maintenance [30, 31]. As part of the implementation, the role of the administration is to assess the feasibility and enforceability of the tax legislation, calculate the implementation costs, translate the legislation into administrative regulations [32], execute the implementation itself and conduct an evaluation. The interpretation of tax laws, based on traditions, ambitions and different administrative interests [32–34], is issued as regulations, decrees and/or general rulings (“executive rules”) by the administration for the implementing authorities even if the final interpretation of tax laws belongs to the judiciary [35, 36].

Based on this very complex situation, the implicit assumption that a tax in law is a tax that is levied because it is an important priority for governments and a key capacity for the development of a functioning state [25, 37] may not always be  proven.

To date, academic attention has mainly been focused on agenda-setting (facilitators and barriers) [22, 38–44] and the impact of taxation on price, purchasing, consumption, revenue streams and other outcomes [45–49]. The actual implementation processes (transposition into administrative regulations, implementation, monitoring and enforcement), however, have rarely been analysed [22, 50–52]. Popki and Ng (2021) argue that SSB taxes are relatively easy to implement from a practical perspective compared to other nutrition policies [53]. However, this argument is not yet well supported, as we know too little about the implementation process.

The aim of this study is to analyse implementation processes for SSB taxation in terms of (1) pre-implementation context, (2) instruments used and (3) interactions in the implementation process. This will in turn allow a better understanding of implementation processes as well as the links between implementation, agenda-setting/formulation, the outcomes and impacts of the SSB tax. Further, a better understanding of the implementation is important to leverage limited resources and safeguard and sustain the impact of the policy [21].

## Methods

The scoping review follows the structured and predefined process of systematic reviews with a focus on identifying and mapping the available evidence about SSB implementation processes [54–57]. To depict the flow of information through the different phases of the scoping review, the Preferred Reporting Items for Systematic Reviews and Meta-Analyses: extension for Scoping Reviews (PRISMA-ScR and flowchart) were used [58]. A study protocol was published beforehand with Open Science Foundation [59].

### Search strategy and information sources

An experienced information specialist in the review team developed and conducted the search strategy. The search structure combined three concepts: SSBs, public policy, and implementation process. Appropriate keywords and their synonyms and controlled vocabulary terms for all relevant terms were used. The search syntax and controlled vocabulary were adapted for subsequent searches in other databases on other platforms. No limits for language, publication date or study design were applied. The search strategy for MEDLINE is available in Additional file 4: Appendix 4.

Structured searches were conducted in the following electronic databases in February 2020: MEDLINE via Ovid (1946–search date); EMBASE via Ovid (1947–search date); PsycINFO via Ovid (1806–search date); Cumulative Index to Nursing and Allied Health Literature (CINAHL) via Ebsco (1981–search date); EconLit via Ebsco (1886–present); Applied Social Sciences Index and Abstracts (ASSIA) via ProQuest (1987–search date); Education Resources Information Center (ERIC) via ProQuest (1966–search date); PAIS via ProQuest (1914–search date); Scopus (1970–search date); and the Social Sciences Citation Index (SSCI) and Science Citation Index–Expanded (SCI-Expanded) 1900–search date; Arts & Humanities Citation Index (A&HCI) 1975–search date; Book Citation Index–Science (BKCI-S) 2013–search date; and Book Citation Index–Social Sciences & Humanities (BKCI-SSH) 2013–search date via Web of Science. Additionally, four sources were searched systematically for grey literature resources: OpenGrey, ThinkTank, and BASE, as well as Google Scholar. The references of included studies as well as previously published reviews and studies were hand-searched for additional citations.

All results were exported to EndNote^®^ reference management software for deduplication. Deduplicated results were imported to Covidence^®^ systematic review management software for title/abstract and full-text screening.

All studies were screened by two reviewers independently following predefined criteria. Any disagreements during the screening and extraction process were resolved by consensus.

### Inclusion and exclusion criteria

All peer-reviewed papers available in full text that reported on the SSB taxation implementation process were included. The inclusion criterion was the reporting of the process of the steps taken after the adoption of the law on taxation of SSB. Papers were included if they examined the process, such as what was done to turn the law into a directive for the implementing authorities, who was tasked with implementation, how implementation was monitored, and how the individual actors (administrative staff, controllers, customs staff, company representatives) were informed. No language or study type restrictions were applied. If an article had to be excluded due to a lack of language proficiency in the review team (German, English, French, Polish), it was marked accordingly.

### Data extraction, coding and analyses

Data were extracted following a previously developed and tested extraction sheet. The extraction was repeated for 10% of the identified studies for quality assurance. Data collected were categorized into the following groups: policy context/pre-implementation context, instrument description, and data characterizing the implementation process itself (Table 2). Further, we collected information about the theoretical frameworks used within the single case studies. Frameworks help to understand and guide the steps involved in the implementation process. Information on frameworks was collected in order to capture which frameworks have been used to study the implementation of tax legislation.

**Table 2:** Categories for data extraction (detailed theoretical reasoning provided in Additional file 2: Appendix 2, Table A.2.1)

Category	Data extracted
Technical details about the paper	Author, country, year, field, administrative level
	Study design
	Framework/theoretical approach mentioned
Policy context	Date of enactment, revision, termination
	Information about development/agenda-setting process (pre-implementation context)
	Aim of the policy
	Reasons for the policy
Instrument description	Type of tax
	Products covered
	Instruments/instrument mix used (communicative, regulatory, economic) One vs bundle of instruments
Implementation process	Events mentioned
	Setting
	Target group behaviour requirement
	Description and relationship of actors (types of organizations involved, positions of the actors, power and hierarchical dependencies)
	Organization of the implementation process
Other	Any further variables reported

Detailed information and the extraction sheet were preregistered in the protocol [59] and can be found with theoretical reasoning and references in Additional file 2: Appendix 2, Table A.2.1. A narrative synthesis of the included studies was used to analyse and interpret the data.

## Results

### Study characteristics

**Fig. 2:**
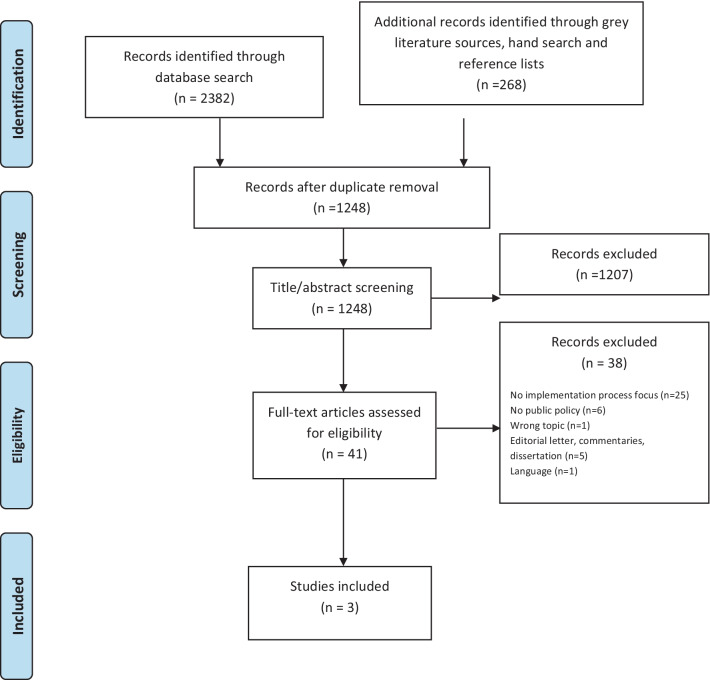
Flow diagram

A total of 2650 publications were identified, of which 1402 were removed as duplicates. The remaining 1248 titles and abstracts were screened; of these, 1207 were excluded, leaving 41 full texts for review. Thirty-eight publications were excluded after full-text screening, leaving three publications for synthesis (Fig. 2). Descriptive details of the papers included are summarized in Additional file 3: Appendix 3.

Three papers, presenting six case studies, were included. They cover implementation processes of SSB taxation in Portugal [60], the implementation of soft drink taxation in the Pacific region (Fiji, Samoa, Nauru, French Polynesia) [61] and the process of tax implementation in the city of Berkeley, California [62]. The jurisdictions covered were state level (*n* = 5) and city level (*n* = 1). Frameworks used to guide the date collection vary between the case studies. For the Portuguese case study, the Health in All Policies framework was used [60]. Thow et al. used a combination of the policy cycle and the Advocacy Coalition Framework for the stakeholder analyses to gain insights into the implementation process in the Pacific region [61]. Falbe et al., addressing the SSB taxation implementation in Berkeley, did not mention a specific framework for their case study [62]. Data reported in included studies were collected via stakeholder interviews and policy document analysis [61], from semi-structured interviews [62] and based on sources not further described [60].

### Policy context

#### Tax enactment, timeline and evolution

In Samoa the tax was introduced in 1984, combined with an import tax in 2007, and both taxes were increased in 2008. In Fiji, Nauru and French Polynesia, taxes were introduced in early 2000 [61]. In Fiji, an import excise duty and an excise duty on locally manufactured products were enacted in 2006, reduced in 2007 due to industry lobbying and replaced by a 3% import duty on raw material [61]. French Polynesia introduced its tax in 2002 and Nauru in 2007 by proposing the tax during a governmental debate on increasing the governmental budget [61]. Portugal enacted its tax in 2017, based on the work of the Portuguese National Programme for the Promotion of Healthy Eating (PNPAS) that started its work in 2012 [60]. The city of Berkeley passed the law in November 2014, and it went into effect in January 2015 [62].

#### Pre-implementation context and use of pre-existing structures

In Portugal and Berkeley, although the implementation was led by the ministries and the municipality, respectively, strong support and involvement of different societal stakeholders for implementation was reported. In Portugal, work began in 2012 with the PNPAS; in September 2016, Council of Ministers Decision No. 334/2016 was promulgated based on the work of the programme. It includes four strategic areas of intervention and proposals for different initiatives/actions [(i) creating a healthier food environment (with taxation as one part), (ii) improving quality and consumer access to healthy food, (iii) promoting and developing consumer competence in healthy food choices, (iv) promoting innovation and entrepreneurship in the field of healthy food promotion] [60]. These have been defined on the basis of proposals submitted by different ministries, are based on the national strategy and are aligned with the WHO European Food and Nutrition Action Plan 2015–2020 and the European Commission's High Level Group on Nutrition and Physical Activity (DG Santé, European Commission). Further experiences and findings from the Portuguese dietary intake survey (2015/2016) were considered. The Portuguese Directorate-General of Health was responsible, as nutrition policy is the responsibility of the Ministry of Health (with the exception of food labelling issues, which is the responsibility of the Ministry of Agriculture) [60]. They coordinated with the Ministry of Finance, Ministry of Internal Affairs, Ministry of Education, Ministry of Health, Ministry of Economy, Ministry of Agriculture and Ministry of the Sea. In addition, stakeholders, including institutions from the public and private sectors as well as civil society, were involved in the agenda-setting process. In 2016, a working group was established to develop an internal ministry structure to promote healthy nutrition, following a “Health in All Policies” or “whole-of-government” approach. In 2017, the Integrated Strategy for the Promotion of Healthy Eating (EIPAS) was initiated by Decree No. 11.418/2017. The EIPAS working group is responsible for monitoring the progress of implementation and submitting biannual reports to the Portuguese government, also using the intra-ministerial group and pre-decree structures [60].

In Berkeley, the city government opted for a tax, but the revenue was to go into a general fund to keep the voting threshold at a simple majority. It was agreed to set up an expert panel to advise the city on funding programmes to further reduce SSB consumption. While the revenue was not earmarked, the panel ensured its use for new public health programmes. In addition, the taxation was supported by various community stakeholders (parents, teachers, health professionals, Latinos and others) prior to its enactment and is based on early leadership engagement in several city departments (Finance, City Attorney’s Office, Public Health, City Manager’s Office) [62].

No data on the pre-implementation context and existing structures were reported in the article covering Samoa, Fiji, Nauru and French Polynesia [61].

### Instrument description

#### Scope of taxation

The objective of the taxes varied. In Portugal and Berkeley, the tax was introduced with the aim of promoting healthier food consumption habits [60, 62]. In French Polynesia, the aim was to generate funds for the establishment of a prevention fund and to finance hospitals [61]. In Nauru, health behaviour and revenue generation were coupled [61]. In Fiji and Samoa, the primary objective was to generate revenue to offset budget deficits due to losses from trade liberalization [61].

#### Tax type

Portugal introduced a specific tax [60]. Nauru used a sugar levy [61]. The city of Berkeley implemented an excise tax [62]. Import and excise taxes were used in Fiji, Samoa and French Polynesia [61].

#### Linkage of instruments

In Portugal, the adoption of the SSB tax was one measure within a set of 51 actions in four priority areas and linked with regulations such as the regulation of unhealthy food marketing to children and regulation of food availability [60]. In Fiji and French Polynesia, the introduction and implementation of the tax was part of a new revenue initiative by the state [61]. In Samoa, it was a stand-alone measure that was later coupled with an import tax [61]. In Nauru, it was a single measure but combined with the removal of bottled water taxation to offset the impact of the tax on household income [61]. In Berkeley it was a single tax initiative [62].

The city of Berkeley and Samoa reported combining regulations with communication tools by conducting information campaigns. Samoa used an educational campaign on the importance of healthy eating before implementing the tax [61]. Part of the city of Berkeley’s implementation plan was an information campaign to increase awareness and the information base of businesses [62]. For the other countries, no other instruments accompanying the tax law were reported.

### Implementation characteristics

#### Implementation structure

The implementation processes were not reported in detail. For Fiji, the Fiji Revenue and Customs Authority is the official tax authority responsible for implementation and tax collection. More detailed procedures and processes between the legislative level and the implementing organization were not reported [61].

Berkeley contracted a tax administration company to coordinate outreach and tax collection, which is overseen by the Sugar-Sweetened Beverage Product Panel of Experts (SSBPPE) [64]. As the city was aware that the departments could not set up the new tax structure as quickly as needed, a tax administration company was hired to coordinate tax collection and outreach (costing 20% of the tax revenue). To support implementation, tax calculation was simplified and an implementation plan including a communication strategy was agreed upon at the outset. In addition, internal staff were recruited to support implementation [62].

In Fiji, Samoa, Nauru and French Polynesia, tax collection was added to the existing mechanisms for tobacco and alcohol. No new structures were created [61].

## Discussion

The aim of this study was to analyse implementation processes for SSB taxation in terms of (1) pre-implementation context, (2) taxation instruments used and (3) interactions in the implementation process. Taxes are used to generate income, but are also instrumental in encouraging or discouraging certain forms of behaviour. However, the use of taxes for instrumental issues tends to complicate tax legislation. That in turn may increase ineffective tax administration [36].

To date, there is little information on processes for implementing SSB taxation. Aside from the work of Falbe et al. [62], Thow et al. [63] and Graca et al. [60], two additional papers analysing SSB taxation implementation have been published since the current work was completed: a policy brief covering a narrative review about the adoption and implementation of SSB taxes in the United States by Chriqui et al. [22], and an implementation report about the SSB tax implementation in Oakland, California, by Asada et al. [21]. Both papers are addressed in the discussion of the results.

The distribution of tax law-making authority differs between centralized (e.g. Samoa) and federal (e.g. United States) governments. In a federal system, the question is how to distribute tax law-making power with respect to major taxes while maintaining an economic and monetary union. In a federal state, both the federal government and the states often have full power to raise important taxes, such as corporate and individual income tax and sales taxes [35]. In addition to law-making power, stakeholders, their access to the policy-making and implementation process and their ability to exert influence play a role. Studies conducted in Berkeley and Portugal reported an implementation based on cooperation and structures developed during the agenda-setting phase. For the Pacific Islands, such a cooperation was not reported.

The cases found in this map are examples of both central and federal states as well as the city government level. With the fragmented data available so far, it is difficult to assess whether the organization of the underlying government system and the distribution of tax authority power have an impact on the implementation of SSB taxes, and if so, what impact and at which critical point of the system [44]. This is of great importance if the tax is used as an instrument to shift behaviour.

It can be assumed that taxation is always a very political issue, especially in the case of SSB, which is controversial both politically and among stakeholders [22]. Political and public support within the implementation process was considered to be supportive. Furthermore, it was reported that structures built during the agenda-setting phase were used within the implementation process (Portugal, city of Berkeley).

The cooperative structures involving civil society actors were not reported for the Pacific Islands. This could be for different reasons: (1) the taxes were attached to existing tax structures and were thus more a technical-formal act, as no new structures were created, (2) the taxes were less controversial or less prominent in the awareness of the actors, or (3) the underlying political system and civil society participation processes are not (yet) designed for this kind of participation. Further work is needed here to gain a better understanding of the role and effects of cooperative structures for SSB tax implementation.

For the Pacific Islands, besides French Polynesia, the aim of the tax was revenue generation for the global governmental budget. Tax collection was linked to existing structures for tobacco and alcohol taxation. No further information about the implementation process was mentioned. The result could be cautiously interpreted in the same direction as results reported by Hagenaars et al. [24], Chiriqui et al. [22] and Asada et al. [21]. Tax capacity in tax levying is important. To monitor and enforce taxation, a certain degree of administrative capacity is necessary. It could be argued that in countries with less developed tax collection mechanisms, collection is linked to existing structures such as alcohol and tobacco taxation.

In Berkeley, a tax collection company was hired. The current data do not allow conclusions to be drawn as to whether or not the use of specialized institutions may impact implementation processes in a positive or negative direction. Outsourcing of highly specialized scientific and technical expertise can be beneficial to the implementation process, as there is no need to build up expensive and time-consuming structures of one’s own [21, 62]. Furthermore, the autonomous position of the agencies vis-à-vis the political decision-making level can lead to effective implementation [64–67]. The hiring of a company in Berkeley was seen as a positive factor in the implementation process [62]. Further, the city government possessed control and enforcement authority and also the possibility to terminate the contract if it was not fulfilled. This concrete programming of implementation reduces the leeway the agency has and increases the pressure to comply with the policy [68].

In Portugal, the implementation process was accompanied by already established oversight and reporting mechanisms, which were set up during the agenda-setting phase. This could have a positive impact on the interpretation of the tax law by the administrative levels and the monitoring and feedback loops that maintain awareness of the tax implementation process within the administrative and political bodies. However, this remains speculative, as no data were reported on this.

Furthermore, it could be argued that by linking SSB tax to budget and revenue questions, the political tensions and conflict lines might be bypassed for some time to favour an administration-based implementation [69]. However, this hypothesis has to be tested by further studies and cannot be generalized at this point. Also, following this line of argument, it should be kept in mind that the lines of conflict may only have been shifted from the political to the administrative level, as the example of the tax modification in Fiji 1 year after its adoption has shown [61]. Additionally, with regard to low- and middle-income countries that have not yet established a robust and efficient tax system and are introducing SSB taxation, linking taxation to existing structures might support implementation, because the regulations and administrative structures are known and no training for staff is needed. However, this action might also be subject to risk [25]. Tax capacity and tax structure depend on the strength and capabilities of the tax administration itself, as tax administrations are complex and require a vast amount of resources that not all states can afford [70].

For Fiji, it was reported that the SSB tax was reduced and modified 1 year after enactment due to industry lobbying [61]. This indicates the possibility for stakeholders to influence legal regulations within administrative processes of implementation [71, 72].

Constant negotiations are necessary to secure funding, as implementation is costly and time-consuming. This opens up opportunities for different stakeholder groups (elected representatives, bureaucrats, industry and other stakeholders) to reopen debates about positions and appropriate solutions that they had not won in the agenda-setting phase, with opportunities to influence the future course of the policy [71]. To reduce the challenges and costs of implementation, implementers will typically seek compromises with such stakeholders [32, 73–75]. Both Chriqui et al. and Asada and colleagues pointed out in their work that after the tax was enacted, the tax legislation must be protected from the influence of anti-SSB tax groups in order to prevent a subsequent amendment of the legislation in their favour [21, 22].

### Limitations

The scoping review has some limitations. Due to the limited number of studies, this is a first look into the “black box” of implementation processes, and a generalization of the results is not possible as there are too few studies focusing on this topic [22, 24, 62]. So far, more than 45 states and jurisdictions have implemented SSB tax laws; however, reports covering the organization and process of the implementation were found in only six cases. Additionally, the information reported is highly fragmented, on both the thematic and the judicial level. More studies are needed that combine agenda-setting and formulation of SSB taxation policies, impact evaluations and implementation studies. This would allow a comparison between policy objectives and actual effects and would enable analysis of the causes of deviation from the objectives. Additionally, a larger database would allow the role of individual variables, such as the use of specialized institutions for tax enhancement or the monitoring capacity of the tax authority, to be isolated and tested in regard to the implementation and the observed outcome and impact of the tax. Further, those studies should be combined with work on stakeholder positions and stakeholder engagement to obtain a broader picture. We used broad theoretical underpinnings to develop the data collection; however, no framework in particular was used to provide guidance, as none really fit. Our study indicates that the work to combine public policy concepts and implementation research frameworks has been initiated and should be continued in the future to inform data collection and increase the comparability of the reported data [21, 76, 77].

## Conclusion

It is important to capture the impact of the SSB tax, but also to understand the underlying political processes, stakeholder interactions and mechanisms that lead to the implementation of the policy. Only by focusing on the implementation process is it possible to link policy goals to their effects. Further, underlying political structures and pre-implementation contexts have an influence on the actual administrative implementation process. Focusing purely on effects is not sufficient to conduct the aim/is comparison and also falls short with regard to safeguarding existing SSB tax laws against anti-tax interference. Here, research on SSB taxation implementation is only at the beginning.

## Supplementary Information

**Additional file 1.** Overview SSB tax worldwide (A.1) and types of taxes used (A.2).

**Additional file 2.** Data extraction sheet and theoretical reasoning.

**Additional file 3.** Detailed study characteristics.

**Additional file 4.** Example of a search strategy for Medline.

## Data Availability

Not applicable.

## Abbreviations

SSBs: Sugar-sweetened beverages
SSBPPE: Sugar-Sweetened Beverage Product Panel of Experts
NNSs: Non-nutritive sweeteners

## References

[1] NCD Risk Factor Collaboration (NCD-RisC) (2016). Trends in adult body-mass index in 200 countries from 1975 to 2014: a pooled analysis of 1698 population-based measurement studies with 19.2 million participants. The Lancet.

[2] The GBD 2015 Obesity Collaborators (2017). Health effects of overweight and obesity in 195 countries over 25 years. N Engl J Med.

[3] DiMeglio DP, Mattes RD (2000). Liquid versus solid carbohydrate: effects on food intake and body weight. Int J Obes.

[4] Mourao DM, Bressan J, Campbell WW, Mattes RD (2007). Effects of food form on appetite and energy intake in lean and obese young adults. Int J Obes.

[5] Malik VS, Hu FB. The role of sugar-sweetened beverages in the global epidemics of obesity and chronic diseases. Nat Rev Endocrinol. 2022. 10.1038/s41574-021-00627-6 PMC8778490 35064240

[6] Ercin AE, Aldaya MM, Hoekstra AY (2011). Corporate water footprint accounting and impact assessment: the case of the water footprint of a sugar-containing carbonated beverage. Water Resour Manag.

[7] Hoekstra AY, Chapagain AK (2007). Water footprints of nations: water use by people as a function of their consumption pattern. Water Resour Manag.

[8] Hoekstra AY (2019). The water footprint of modern consumer society.

[9] Brownell KD, Farley T, Willett WC, Popkin BM, Chaloupka FJ, Thompson JW, Ludwig DS (2009). The public health and economic benefits of taxing sugar-sweetened beverages. N Engl J Med.

[10] Chaloupka FJ, Powell LM, Warner KE (2019). The use of excise taxes to reduce tobacco, alcohol, and sugary beverage consumption. Annu Rev Public Health.

[11] Implementing taxes on sugar-sweetened beverages: an overview of current approaches and the potential benefits for children. https://sunpc.org.pk/wp-content/uploads/2019/05/190328_UNICEF_Sugar_Tax_Briefing_R09.pdf; 24.02.2021.

[12] Baker P, Jones A, Thow AM (2018). Accelerating the worldwide adoption of sugar-sweetened beverage taxes: strengthening commitment and capacity comment on “the untapped power of soda taxes: incentivizing consumers, generating revenue, and altering corporate behavior”. Int J Health Policy Manag.

[13] Bandy LK, Scarborough P, Harrington RA, Rayner M, Jebb SA (2020). Reductions in sugar sales from soft drinks in the UK from 2015 to 2018. BMC Med.

[14] Reyes M, Smith Taillie L, Popkin B, Kanter R, Vandevijvere S, Corvalán C (2020). Changes in the amount of nutrient of packaged foods and beverages after the initial implementation of the Chilean Law of Food Labelling and Advertising: a nonexperimental prospective study. PLOS Med.

[15] Le Bodo Y, De Wals P (2018). Soda taxes: the importance of analysing policy processes comment on “the untapped power of soda taxes: incentivising consumers, generating revenue, and altering corporate behaviours”. Int J Health Policy Manag.

[16] Roache SA, Gostin LO (2017). The untapped power of soda taxes: incentivizing consumers, generating revenue, and altering corporate behavior. Int J Health Policy Manag.

[17] Allcott H, Lockwood BB, Taubinsky D (2019). Should we tax sugar-sweetened beverages? an overview of theory and evidence. J Econ Perspect.

[18] Global Food Research Program. Sugary drink taxes around the world. Chapel Hill; 2020.

[19] Prevention: Tax and pricing. https://www.obesityevidencehub.org.au/privacy; 30.11.2020.

[20] World Bank (2020). Taxes on sugar-sweetened beverages: international evidence and experiences.

[21] Asada Y, Chriqui JF, Pipito AA, Taher S, Powell LM (2022). “Holding the City’s feet to the fire”: lessons learned from Oakland’s implementation of measure HH sugar-sweetened beverage tax. J Public Health Manag Pract.

[22] Chriqui JF, Pipito AA, Asada Y, Powell LM. Lessons learned from the adoption and implementation of sweetened beverage taxes in the United States: a narrative review. Policy, Practice and Prevention Research Center, University of Illinois, Chicago, Chicago, IL: Research Brief No.119; 2021.

[23] Le Bodo Y, Paquette M-C, De Wals P (2016). Taxing soda for public health: a Canadian perspective.

[24] Hagenaars LL, Jeurissen PPT, Klazinga NS (2017). The taxation of unhealthy energy-dense foods (EDFs) and sugar-sweetened beverages (SSBs): an overview of patterns observed in the policy content and policy context of 13 case studies. Health Policy.

[25] Pomeranz D, Vila-Belda J (2019). Taking state-capacity research to the field: insights from collaborations with tax authorities. Ann Rev Econ.

[26] Besley T, Persson T, Auerbach A, Chetty R, Feldstein M, Saez E (2013). Public finance and development. Handbook of public economics.

[27] Herbst J (1990). War and the State in Africa. Int Secur.

[28] Slemrod J (2007). Cheating ourselves: the economics of tax evasion. J Econ Perspect.

[29] Slemrod J (2019). Tax compliance and enforcement. J Econ Lit.

[30] Howlett M (2019). Designing public policies: principles and instruments.

[31] Knill C, Tosun J. Policy Making. In: Caramani D. (eds.). Comparative politics. Oxford: Oxford University Press; 2008: 495–519.

[32] Perl A, Capano G, Howlett M (2020). Studying policy dynamics: policy cycles and regimes. A modern guide to public policy.

[33] Bardach E (1977). The implementation game: what happens after a bill becomes a law.

[34] Elmore RF (1978). Organizational models of social program implementation. Public Policy.

[35] Vanistendael V, Thuronyi V (1996). Legal framework for taxation. Tax law design and drafting.

[36] van Kommer V, Alink M. Chapter 6: The process of tax policymaking and legislation. In: van Kommer V, Alink M (eds.). The Dutch approach description of Dutch tax and customs administration. Vol 3rd. IBFD; 2012: 61–78

[37] Besley T, Persson T (2009). The origins of state capacity: property rights, taxation, and politics. Am Econ Rev.

[38] Jou J, Techakehakij W (2012). International application of sugar-sweetened beverage (SSB) taxation in obesity reduction: factors that may influence policy effectiveness in country-specific contexts. Health Policy.

[39] Purtle J, Langellier B, Lê-Scherban F (2018). A case study of the Philadelphia sugar-sweetened beverage tax policymaking process: implications for policy development and advocacy. J Public Health Manag Pract.

[40] James E, Lajous M, Reich MR (2020). The politics of taxes for health: an analysis of the passage of the sugar-sweetened beverage tax in Mexico. Health Syst Reform.

[41] Karim SA, Kruger P, Hofman K (2020). Industry strategies in the parliamentary process of adopting a sugar-sweetened beverage tax in South Africa: a systematic mapping. Glob Health.

[42] Hilton S, Buckton CH, Patterson C, Katikireddi SV, Lloyd-Williams F, Hyseni L, Elliott-Green A, Capewell S (2019). Following in the footsteps of tobacco and alcohol? Stakeholder discourse in UK newspaper coverage of the Soft Drinks Industry Levy. Public Health Nutr.

[43] Chriqui JF, Sansone CN, Powell LM (2020). The sweetened beverage tax in Cook County, Illinois: lessons from a failed effort. Am J Public Health.

[44] Hagenaars LL, Jevdjevic M, Jeurissen PPT, Klazinga NS (2020). Six lessons from introducing sweetened beverage taxes in Berkeley, Cook County, and Philadelphia: a case study comparison in agenda setting and decision making. Health Policy.

[45] Teng AM, Jones AC, Mizdrak A, Signal L, Genç M, Wilson N (2019). Impact of sugar-sweetened beverage taxes on purchases and dietary intake: systematic review and meta-analysis. Obes Rev.

[46] The evidence on the effects of soft drink taxes. https://www.ifs.org.uk/uploads/BN255-the-evidence-on-the-effects-of-soft-drink-taxes.pdf; 24.02.2021.

[47] Grummon AH, Roberto CA, Krieger JW (2020). Is the association between beverage taxes and reductions in sales driven by communication of health consequences in addition to price increases?. JAMA Netw Open.

[48] Castelló JV, Casasnovas GL (2020). Impact of SSB taxes on sales. Econ Human Biol.

[49] Falbe J, Thompson HR, Becker CM, Rojas N, McCulloch CE, Madsen KA (2016). Impact of the Berkeley excise tax on sugar-sweetened beverage consumption. Am J Public Health.

[50] Kim D, Kawachi I (2006). Food taxation and pricing strategies to “thin out” the obesity epidemic. Am J Prev Med.

[51] Onagan FCC, Ho BLC, Chua KKT (2019). Development of a sweetened beverage tax Philippines. Bull World Health Organ.

[52] Barquera S, Campos I, Rivera JA (2013). Mexico attempts to tackle obesity: the process, results, push backs and future challenges. Obes Rev.

[53] Popkin BM, Ng SW (2021). Sugar-sweetened beverage taxes: lessons to date and the future of taxation. PLOS Med.

[54] Higgins JPT, Green S, Higgins JPT, Green S (2010). Guide to the contents of a Cochrane protocol and review. Cochrane handbook for systematic reviews of interventions.

[55] James KL, Randall NP, Haddaway NR (2016). A methodology for systematic mapping in environmental sciences. Environ Evid.

[56] Munn Z, Peters MDJ, Stern C, Tufanaru C, McArthur A, Aromataris E (2018). Systematic review or scoping review? Guidance for authors when choosing between a systematic or scoping review approach. BMC Med Res Methodol.

[57] Reisch LA, Andor MA, Doebbe F, Haddaway NR, Meier J. Mitigating climate change in food consumption and food waste: a systematic map of behavioural interventions, Search Protocol for a Systematic Mapping Study. Copenhagen/Stockholm/Essen: OSF; 2019.

[58] Tricco A, Lillie E, Zarin W, O’Brien K, Colquhoun H, Levac D, Moher D, Peters M, Horsley T, Weeks L (2018). PRISMA extension for scoping reviews (PRISMAScR): checklist and explanation. Ann Intern Med.

[59] Forberger S, Luszczynska A, Lien N, Meshkovska B, Łobczowska K, Scheller D, Wendt J, Christianson L, Reisch L, Zeeb H. Analyzing public health policy implementation processes—a systematic map. osf.io/7w84q: OSF; 2020.

[60] Graca P, Gregorio MJ, de Sousa SM, Bras S, Penedo T, Carvalho T, Bandarra NM, Lima RM, Simao AP, Goiana-da-Silva F (2018). A new interministerial strategy for the promotion of healthy eating in Portugal: implementation and initial results. Health Res Policy Syst.

[61] Thow AM, Quested C, Juventin L, Kun R, Khan A, Swinburn B (2011). Taxing soft drinks in the Pacific: implementation lessons for improving health. Health Promot Int.

[62] Falbe J, Grummon AH, Rojas N, Ryan-Ibarra S, Silver LD, Madsen KA (2020). Implementation of the First US sugar-sweetened beverage tax in Berkeley, CA, 2015–2019. Am J Public Health.

[63] Thow AM, Downs SM, Mayes C, Trevena H, Waqanivalu T, Cawley J (2018). Fiscal policy to improve diets and prevent noncommunicable diseases: from recommendations to action. Bull World Health Organ.

[64] Hawkins DG, Lake DA, Nielson DL, Tierney MJ (2006). Delegation under anarchy: states, international organizations, and principal-agent theory. Deleg Agency Int Organ.

[65] Gilardi F, Braun D (2002). Delegation aus der Sicht der Prinzipal-Agent-Theorie. Politische Vierteljahresschrift.

[66] Pollack MA (2003). The engines of European integration: delegation, agency, and agenda setting in the EU.

[67] Knill C, Tosun J. Einführung in die Policy-Analyse. utb; 2015.

[68] Bogumil J, Jann W (2009). Verwaltung und Verwaltungswissenschaft in Deutschland. 2., völlig überarb. Auflage.

[69] Matland RE (1995). Synthesizing the implementation literature: the ambiguity-conflict model of policy implementation. J Public Adm Res Theory.

[70] Bird RM (2012). Taxation and development: what have we learned from fifty years of research?.

[71] Nicholson-Crotty S (2005). Bureaucratic competition in the policy process. Policy Stud J.

[72] Nestle M. Food politics: how the food industry influences nutrition and health. Univ of California Press; 2013.

[73] Giuliani M, Grant W, Perl A, Knoepfel P (1999). ‘Soft’ institutions for hard problems: instituting air pollution policies in Three Italian Regions. The politics of improving urban air quality.

[74] Hood C (1983). Using bureaucracy sparingly. Public Adm.

[75] Hood C (1986). The tools of government.

[76] Lobczowska K, Banik A, Brukalo K, Forberger S, Kubiak T, Romaniuk P, Scheidmeir M, Scheller DA, Steinacker JM, Wendt J (2022). Meta-review of implementation determinants for policies promoting healthy diet and physically active lifestyle: application of the Consolidated Framework for Implementation Research. Implement Sci.

[77] Lobczowska K, Banik A, Romaniuk P, Forberger S, Kubiak T, Meshkovska B, Neumann-Podczaska A, Kaczmarek K, Scheidmeir M, Wendt J (2022). Frameworks for implementation of policies promoting healthy nutrition and physically active lifestyle: systematic review. Int J Behav Nutr Phys Act.

