# Supplementary material for: Sugar-sweetened beverage tax implementation processes: results of a scoping review
Source: Health Res Policy Syst. 2022 Mar 24;20:33. doi: 10.1186/s12961-022-00832-3 (PMC8944035; doi:10.1186/s12961-022-00832-3)
Supplement: Supplementary file 1 — Additional file 1. Overview SSB tax worldwide (A.1) and types of taxes used (A.2). [file 12961_2022_832_MOESM1_ESM.docx]

# Appendix 1: Overview SSB tax worldwide (A.1) and types of taxes used (A.2)

# Table A.1: Overview SSB worldwide taxes (April 2021)

|  | **Country** | **Year of enactment** | **Tax jurisdiction (local, subnational, nation)** | **Tax type** | **Type of measurement** | **Tax design** | **Tax rate** | **Tax scope** | **Exception** |
| --- | --- | --- | --- | --- | --- | --- | --- | --- | --- |
|  | **North America** | |  |  |  |  |  |  |  |
| **1** | Albany, CA | 2017 | Local (City) | Excise | Specific | Volume based | 1 cent per ounce | on drinks with added caloric sweetener; exempts milk-based drinks, 100% fruit juice | beverages distributed from retailers with revenue <$US 100 000 per annum |
| **2** | Berkeley, CA | 2015 | Local (City) | Excise | Specific | Volume based | 1 cent per ounce | sweetened drinks | meal-replacement, dairy drinks, diet sodas, fruit juice, alcohol |
| **3** | Boulder, CO | 2017 | Local (City) | Excise | Specific | Volume based | 2 cents per ounce | on beverages with ≥ 5 g added caloric sweeteners/12 oz | milk-based drinks and 100% juice |
| **4** | Navajo Nation | 2015 | Local | Excise | Ad valorem |  | 2% junk food tax | “minimal-to-no nutritional value food items,” including sugar-sweetened beverages |  |
| **5** | Oakland, CA | 2017 | Local (City) | Excise | Specific | Volume based | 1 cent per ounce | drinks with added caloric sweeteners | milk-based drinks, 100% juice; beverages distributed from retailers with revenue <$US 100,000 per annum |
| **6** | Philadelphia, PA | 2018 | Local (City) | Excise | Specific | Volume based | 1.5 cents per ounce | sugar-and artificially-sweetened drinks, including diet soda; | milk-based drinks, 100% juice |
| **7** | San Francisco, CA | 2018 | Local (City) | Excise | Specific | Volume based | 1 cent per ounce | drinks with added sugar and >25 kcal per 12 oz; applies to syrup and powder concentrates | 100% juice, artificially sweetened beverages, infant formula, milk products, medical drinks, alcoholic beverages |
| **8** | Seattle, WA | 2018 | Local (City) | Excise |  |  | 1.75 cents per ounce | sugary drinks | diet sodas, milk-based drinks & 100% fruit juice |
|  | **Central** **and South America & Caribbean** | | |  |  |  |  |  |  |
| **9** | Barbados | 2015 | National | Excise | Ad valorem |  | 10% | sugary drinks, including carbonated soft drinks, juice drinks, sports drinks | 100% juice, coconut water, and plain milk |
| **10** | Bermuda | 2019, revised 2018 | National | Import | Ad valorem |  | 75% | sugar, sugary drinks, candies, dilutables | diet sodas, 100% juice, and diet iced teas |
| **11** | Chile | 2014 | National | Excise | Ad valorem |  | 18%, 10% | 18%: sugary drinks containing >6.25 g sugar/100 mL; includes all non-alcoholic drinks with added sweeteners  10%: drinks with <6.25 g sugar/100 mL | 100% fruit juice and dairy-based beverages |
| **12** | Dominica | 2015 | National | Excise | Ad valorem |  | 10% | food and drinks with high sugar content, including soft drinks and energy drinks |  |
| **13** | Ecuador | 2016 | National | Excise | Ad valorem |  | 10% | soft drinks with <25g of sugar/L and on all energy drinks, regardless of sugar content; $0.0018 per gram sugar on drinks with >25 g sugar/L | dairy products and their derivatives, mineral water and juices that have 50% of natural content |
| **14** | Mexico | 2014 | National | Excise | Specific | Volume based | 1 peso per litre | drinks with added sugar | Milk, yogurts |
| **15** | Panama | 2019 | National | Excise | Ad valorem |  | 7%, 5%, 10% | 7% on sodas, 5% on other non-alcoholic drinks with added caloric sweeteners; 10% on syrups & concentrates |  |
| **16** | Peru | 2018, revised 2019 | National | Excise | Ad valorem |  | 25%, 17%, 12% | 25% on drinks containing >6 g sugar/100 mL (increase from 17%); 17% ad valorem tax on drinks with 0.5–6 g sugar/100 mL (unchanged); 12% ad valorem tax on drinks with <0.5 g sugar/100 mL (decrease from 17%). | Plain water, 100% juice, plain milk, drinkable yogurts |
|  | **Europe** |  |  |  |  |  |  |  |  |
| **17** | Belgium | 2016 | National | Excise | Specific | Volume based | €0.068/L, €0.41/L,€0.068/100kg | €0.068/L: soft drinks with added sweeteners  €0.41/L and €0.68/100 kg (powders) excise on concentrates |  |
| 18 | Catalonia, Spain | 2017 | Subnational | Excise | Specific | Tiered volume based | €0.12/L, €0.8/L | €0.12/L: drinks with added sugars and >8 g sugar/100 mL  €0.08/L: 5–8 g sugar/100 mL |  |
| 19 | Finland | 1940, revised 2011 | National | Excise | Specific | Volume based | €0.22/L, €0.12/L | €0.22/L: sugar-containing soft drinks  €0.12/L: sugar-free soft drinks, mineral waters |  |
| 20 | France | 2012, revised 2018 | National | Excise | Specific | Sugar based | €0.11/1.5L, €20/hL | €0.11/1.5L: drinks with added sugars or artificial sweeteners  Sliding scale tax, up to €20/ hL: if >11g sugar/100mL |  |
| 21 | Hungary | 2011 | National | Excise | Specific | Volume based | HUF 7/L, HUF 200/L | HUF 7/L: soft drinks  HUF 200/L: syrup concentrates |  |
| 22 | Ireland | 2018 | National | Excise | Specific | Tiered volume based | €0.20/L, €0.030/L | €0.20/L: drinks with >5 g total sugar/100 mL  €0.30/L: on drinks with >8 g total sugar/100 mL |  |
| 23 | Latvia | 2004, revised 2016 | National | Excise | Specific | Volume based | €0.074/L | drinks with added sugar, sweetener, or other flavouring | fruit/vegetable juices with <10% added sugar and flavoured/functional waters without added sugars, sweeteners, or flavourings |
| 24 | Norway | 1981 | National | Excise | Specific | Volume based | NOK 3.34/L, NOK 20.32/L | NOK 3.34/L: drinks containing added sugar or sweeteners  NOK 20.32/L: syrup concentrates |  |
| 25 | Poland | 2021 | National | Excise |  |  | PLN 0.5/L, PLN 0.09/L, PLN 0.05 extra fee | PLN 0.5/L: soft drinks with added sweeteners, caffeine, or taurine;  PLN 0.09/L: drinks containing caffeine or taurine.  PLN 0.05 extra fee per gram of sugar>5 g/100 mL;  Total soda fee cannot exceed PLN 1.2. | sports or juice drinks with <5g sugar/100mL and dairy drinks. Drinks with >20% juice content and >5g sugar/100mL are not charged the base fee of PLN 0.5 per L |
| 26 | Portugal | 2017 | National | Excise | Specific | Tiered volume based | €0.08/L, €0.16/L | €0.08/L: drinks with a sugar content of <80 g/L  €0.16/L: drinks with >80 g/L sugar |  |
| 27 | Spain | 2021 | National | VAT |  |  | 21% | drinks containing added natural and derived sweeteners and/or sweetening additives | dairy |
| 28 | St. Helena | 2014 | National | Excise | Specific | Volume based | £0.75/L | carbonated drinks with ≥15 g sugar/L |  |
| 29 | United Kingdom | 2018 | National | Excise | Specific | Tiered volume based | £0.18/L, £0.24/L | £0.18/L: drinks with >5 g total sugar/100 mL  £0.24/L: on drinks with >8 g total sugar/100 mL |  |
|  | **Asia & Pacific** |  |  |  |  |  |  |  |  |
| 30 | Brunei | 2017 | National | Excise | Specific | Tiered volume based | BND 4.00/L | all drinks with >6 g sugar per 100mL |  |
| 31 | Cook Islands | 2013, revised 2014 | National | Excise | Specific | Sugar based | Beverages containing  added sugars | Artificially sweetened waters |  |
| 32 | Fr. Polynesia | 2002 |  | Local tax, import tax | Specific | Volume based | CFP 40/L local, CFP 60/L import | sweetened drinks |  |
| 33 | Fiji | 2007, revised 2016, 2017 | National | Local tax, import | Specific (excise), ad valorem (import) | Specific: volume based | FJD0.25/L local, 15% , 10#%import duty | 15%: on sweetend drinks  10%: concentrates |  |
| 34 | India | 2017 | National | Goods & services tax (GST) |  |  | 12% good & services, 28% GST | 12%: all processed packaged beverages and foods  28% GST: additional on aerated beverages and lemonades |  |
| 35 | Kiribati | 2014 | National | Excise | Ad valorem |  | 40% | drinks containing added sugar and fruit concentrates | 100% juices |
| 36 | Malaysia | 2019 | National | Excise | Specific | Tiered volume based | RM 0.40/L | carbonated, flavoured, & other non-alcoholic drinks with >5 g sugar per 100 mL or on fruit or vegetable juices with >12 g sugar per 100 mL |  |
| 37 | Maldives | 2017 | National | Import |  |  | MVR 33.64/L, MVR 4.60/L | MVR 33.64/L: all energy drinks  MVR 4.60/L: soft drinks (incl. sweetened and unsweetened carbonated sodas, sports drinks) |  |
| 38 | Nauru | 2007 | National | Import | Ad valorem |  | 30% | all products with added sugars (+ removal of bottled water levy) |  |
| 39 | Palau | 2003 | National | Import | Specific | Volume based | $0.28175/L | carbonated soft drinks |  |
| 40 | Philippines | 2018 | National | Excise | Specific | Volume based | 6 pesos/L, 12 pesos/L | 6 pesos/L: drinks using sugar and artificial sweeteners  12 pesos/L: drinks using HFCS | dairy drinks, sweetened instant coffee, drinks sweetened using coco sugar or stevia, and 100% juices |
| 41 | Samoa | 1984, revision 2007 | National | Excise, import | Ad valorem |  | WST 0.40/L | carbonated beverages |  |
| 42 | Thailand | 2017 | National | Excise | Specific | Tiered volume based,  Ad valorem | 2023 onward:  6–8 g/100 ml - THB 1/L  8–10 g/ 100 ml - THB 3/L  >10 g/ 100 ml - THB 5/L | Artificial mineral water, soda water, carbonated soft drinks without sugar  or other sweeteners and without flavour, mineral water and carbonated  soft drinks with added sugar or other  sweeteners or flavours, and fruit and vegetable juices |  |
| 43 | Tonga | 2013, revised 2016 | National | Excise | Specific | Volume based | 1 Pa’anga/L | carbonated beverages |  |
| 44 | Vanuatu | 2015 | National | Excise | Specific | Volume based | 50 vatu/L | carbonated beverages containing added sugar or other sweeteners |  |
|  | **Africa, Middle East** |  |  |  |  |  |  |  |  |
| 45 | Bahrain | 2017 | National | Excise | Ad valorem |  | 100%, 50% | 100%: energy drinks  50%: aerated soft drinks |  |
| 46 | Mauritius | 2013, revised 2016 | National | Excise | Specific | Tiered volume based | MUR 0.03/g sugar | sodas, syrups, and fruity drinks with added sugar |  |
| 47 | Morocco | 2019 | National | VAT |  |  | MAD 0.7/L, 0.6/L, 0.15/L, 0.45/L | MAD 0.7/L:soft and non-carbonated drinks with ≥5 g sugar per 100 mL  MAD 0.6/L: energy drinks  MAD 0.15/L: nectars  MAD 0.45/L: soft drink manufacturers |  |
| 48 | Qatar | 2019 | National | Excise | Ad valorem |  | 100%. 50% | 100%: energy drinks  50%: sweetened aerated drinks and concentrates to make carbonated drinks |  |
| 49 | Oman | 2019 | National | Excise | Ad valorem |  | 100% excise, 50% tax | 100%: energy drinks  50%: all carbonated drinks | sparkling water |
| 50 | Saudi Arabia | 2017, revised 2019 | National | Excise |  |  | 100% excise, 50% tax | 100%: energy drinks  50%: sweetened drinks |  |
| 51 | Seychelles | 2019 | National | Import | Specific | Volume based | SCR 4/L | all beverages containing >5 g sugar/100 mL | fresh, locally produced drinks without additives and plain milks |
| 52 | South Africa | 2018 | National | Excise | Specific | Sugar based | ZAR0.021/g sugar | sugary drinks and concentrates (4g per 100mL exempt). If sugar not labelled, default tax based on 20 g sugar/100mL | sugary drinks and concentrates (4g per 100mL), dairy drinks and fruit, vegetable juices |
| 53 | United Arab Emirates | 2017, revised 2019 | National | Excise, tax | Ad valorem |  | 100% excise, 50% tax | 100%: energy drinks  50%: any drinks with added sugar or sweeteners |  |

<https://globalfoodresearchprogram.web.unc.edu/wp-content/uploads/sites/10803/2021/04/SSB_sugary_drink_taxes_maps.pdf> (access: 13.04.2021)

<https://www.obesityevidencehub.org.au/collections/prevention/countries-that-have-implemented-taxes-on-sugar-sweetened-beverages-ssbs> (access: 13.04.2021)

<https://openknowledge.worldbank.org/bitstream/handle/10986/33969/Support-for-Sugary-Drinks-Taxes-Taxes-on-Sugar-Sweetened-Beverages-Summary-of-International-Evidence-and-Experiences.pdf?sequence=6&isAllowed=y> (access: 13.04.2021)

Table A.2: Types of SSB taxes (1)

| **Instrument** | **Description** |
| --- | --- |
| **Excise tax** | Tax levied on a particular product, typically at the point of manufacture or distribution. Can be either *specific* (based on volume or sugar content) or *ad valorem* (based on percentage of product value). *Tiered* and *sliding-scale* designs apply different specific tax rates depending on volume or sugar content. |
| **Import tax (import tariff, import duty, customs duty)** | Tax collected on imported products |
| **VAT/GST** | A VAT is a broad-based tax assessed incrementally as a percentage of price at each stage in the production and distribution chain. Considered a consumption tax because the ultimate cost of paying the tax is borne by the consumer at the point of purchase. A GST is, in most cases, a type of VAT. |

1. World Bank. Taxes on Sugar-Sweetened Beverages: International Evidence and Experiences. Group WB, editor. Washington DC: World Bank; 2020.
