# Supplementary material for: Sugar-sweetened beverage tax implementation processes: results of a scoping review
Source: Health Res Policy Syst. 2022 Mar 24;20:33. doi: 10.1186/s12961-022-00832-3 (PMC8944035; doi:10.1186/s12961-022-00832-3)
Supplement: Supplementary file 2 — Additional file 2. Data extraction sheet and theoretical reasoning. [file 12961_2022_832_MOESM2_ESM.docx]

**Appendix 2**

**Table A2.1: Detailed data extraction sheet and theoretical reasoning**

| **Type of Data** | **No.** | **Category** | **Characteristics** |
| --- | --- | --- | --- |
| Publication metadata | 1 | Author | Authors of the publication |
|  | 2 | Year | Year of publication |
|  | 3 | Title | Title of the paper |
|  | 4 | Journal | Name of the journal/publication medium |
|  | 5 | Publication type | Journal article, report, theses, book |
| Public policy metadata | 6 | Domain/Subdomain (Jurisdiction) | EU, national, regional, city level |
|  | 7 | Origin | Country of origin |
|  | 8 | Health policy field | SSB, PA, active transport with further specification if mentioned |
|  | 9 | Author of the policy | Name of the public body |
|  | 10 | Date of effect of the policy mentioned | If indicated |
|  | 11 | Date of revision or information about history/evolution of the policy | - If a revision took place  - If the policy is built upon previous policies/programmes/interventions (if the policy has a history) (1, 2) |
|  | 12 | Date of end of validity | If indicated |
|  | 13 | Aim of the policy | Specified in the publication |
|  |  |  |  |
| Data of the study | 14 | Study design | Design used for the analysing the policy |
|  | 15 | Theoretical approach | Theory, framework, model used within the publication |
| Policy/intervention descriptions | 16 | Operationalisation | Is the policy operationalised as one intervention or is it divided into several sub-interventions? Operationalisation of the policy according to:  (a) Target group  (b) Setting  (c) Policy instruments |
|  | 17 | Events | Are specific events mentioned in the connection with the policy (range from elections to disasters to emergent technologies to revolutions)? |
|  | If the policy is operationalised in several sub-interventions, the following points should be analysed per sub-intervention | | |
|  | 18 | Setting | General population, specific settings mentioned within the policy |
|  | 19 | Target group | While the policy targets the whole population, within the operations, special target groups might be mentioned within the policy |
|  | 20 | Instruments used within the policy | Instruments used within the policy/for operationalised interventions as part of the policy (3):   1. Communicative (Information: media transmission, interpersonal transmission) 2. Regulatory (prohibition: unconditional, conditional (with exceptions, with permissions, with obligations; soft law/regulations) 3. Economic means (incentives, disincentives, in kind) |
|  | 21 | Dimension of implementation process | (a) Implementation structure (4-7)   - Type and organizations involved in the process - Organizational setting (horizontal vs vertical, multilevel (how many levels and which), cross-sectional, private actor involvement) - Policy-relates rules, procedures, resource allocation   (b) Agency decision-making (transfer/operationalisation of legal stipulation in operational programmes/interventions) (4, 8)   - who within the process decided on delivery mode, leeway for discretion, scope and nature of involved personnel - information about who guides and drives the process - position of the actors involved (supportive, rejecting, neutral, mixed) (9-11) - information about organisation of the process   (c) Role of the target group within the implementation process e.g. filling out requests, complying with conditions attached to benefits, comply with special treatment programmes (12, 13)  (d) Policy results per intervention and for the whole policy (14) |
|  | 22 | Further variables mentioned/analysed and their operationalisation | Further variables mentioned |
|  | 23 | Evaluation | Is an evaluation planned of the policy? Was there an evaluation of the policy? |
|  | 24 | Outcome/Impact | Results of the policy |

**References**

1. Greer SL. Choosing paths in European Union health services policy: a political analysis of a critical juncture. Journal of European Social Policy. 2008;18(3):219-31.

2. Weible CM, Carter DP. Advancing Policy Process Research at Its Overlap with Public Management Scholarship and Nonprofit and Voluntary Action Studies. Policy Studies Journal. 2017;45(1):22-49.

3. Bemelmans-Videc M, Rist CR, Verdung E. Carrots, Sticks, and Sermons: Policy Instruments and Their Evaluation. 4th ed. New Brunswick, London: Transaction Publishers; 2007.

4. Tosun J, Treib O. Linking policy design and implementation styles. In: Howlett M, Mukherjee I, editors. The Routledge handbook of policy design: Routledge London; 2018.

5. Tosun J. Pursuing Horizontal Management: The Politics of Public Sector Coordination. B. Guy Peters. Lawrence: University Press of Kansas, 2015. 216 pp. $50 (cloth). Governance. 2016;29(4):583-4.

6. Bolleyer N, Börzel TA. Non-hierarchical policy coordination in multilevel systems. European Political Science Review. 2010;2(2):157-85.

7. Thomann E, Lieberherr E, Ingold K. Torn between state and market: Private policy implementation and conflicting institutional logics. Policy and Society. 2016;35(1):57-69.

8. Sager F, Thomann E, Zollinger C, van der Heiden N, Mavrot C. Street-level Bureaucrats and New Modes of Governance: How conflicting roles affect the implementation of the Swiss Ordinance on Veterinary Medicinal Products. Public Management Review. 2014;16(4):481-502.

9. Tsebelis G. Veto players: How political institutions work. Princeton: Princeton University Press; 2002.

10. Haverland M. National Adaption to European Integration: The Importance of Institutional Veto Points. Journal of Public Policy. 2000;20(1):83-103.

11. Steunenberg B. A Policy Solution to the European Union’s Transposition Puzzle: Interaction of Interests in Different Domestic Arenas. West European Politics. 2007;30(1):23 – 49.

12. Howlett M. What is a policy instrument? Tools, mixes, and implementation styles. Designing government: From instruments to governance. 2005:31-50.

13. Pierce JJ, Siddiki S, Jones MD, Schumacher K, Pattison A, Peterson H. Social construction and policy design: A review of past applications. Policy Studies Journal. 2014;42(1):1-29.

14. Mettler S, SoRelle M. Policy feedback theory. In: Weible CM, Sabatier PA, editors. Theories of the policy process. 4 ed. New Yourk: Routledge; 2018. p. 151-81.
