# Supplementary material for: Sugar-sweetened beverage tax implementation processes: results of a scoping review
Source: Health Res Policy Syst. 2022 Mar 24;20:33. doi: 10.1186/s12961-022-00832-3 (PMC8944035; doi:10.1186/s12961-022-00832-3)
Supplement: Supplementary file 3 — Additional file 3. Detailed study characteristics. [file 12961_2022_832_MOESM3_ESM.docx]

# Appendix 3

| **Table 1: Detailed study characteristics as reported in the studies** | | | | | | | | | | | | | | | | | | | | | | | | | | | | | |
| --- | --- | --- | --- | --- | --- | --- | --- | --- | --- | --- | --- | --- | --- | --- | --- | --- | --- | --- | --- | --- | --- | --- | --- | --- | --- | --- | --- | --- | --- |
| **No.** | **Author** | **Year** | **Country** | **Administrative level** | **Health policy field** | **Study design** | **framework/theoretical approach** | **Date of enactment** | **Date of revisions, information about history** | **Termination** | **Aim of the policy** | **Reasons for policy** | **Type of tax** | **Products covered** | **One policy or several sub-interventions (packages)** | **Are events mentioned** | **Setting** | **Target group** | **Communicative** | **Regulatory** | **economic means** | **Type/organizations involved** | **horizontal vs. Vertical implementation** | **who decides** | **position of the actors** | **role of target group (complying requests)** | **Organisation of the implementation** | **Outcome** | **indirect effects** |
| 1 | Graca et al. | 2018 | Portugal | national | SSB | case study | health in all policies | 2017 | 2012, Portugal National Programme for the Promotion of Healthy Eating (PNPAS); four strategic areas of intervention and proposals of different initiatives/actions related to strategic axis defined based on the proposals submitted by different ministries, based on the national strategy and aligned with WHO European Food and Nutrition Action Plan 2015–2020 and the High Level Group on Nutrition and Physical Activity of the European Commission (DG Santé, European Commission) experiences and evidence within the Portuguese dietary intake survey (2015/2016) |  | to encourage healthier food consumption habits as well as to improve the nutritional status of the Portuguese population | NCD driven (obesity rates) | Specific excise tax 16.46€ per hectolitre is applied for soft drinks that contain ≥ 8 g of sugar per 100 mL and a tax of 8.22€ is applied for those with < 8 g of sugar content per 100 mL | all SSBs, referring to any drink with added sugar or other sweeteners  excluded drinks: considered as having nutritional value such as milk or “dairy alternative beverages (e.g., soy, rice, oat, almond, hazelnut, coconut), fruit juices, and drinks considered food for special dietary needs or nutritional supplements, alcoholic drinks | one in a bundle of actions (51 in 4 priority areas) | no | state | population |  | x |  | Ministries   during Preparation phase (2012-2017) inclusion of wide range of stakeholder (industry, research, society) within the discussion process | top-down but with stakeholder participation | Directorate-General of Health | Ministry of Finance, Internal Affairs, Education, Health, Economy, Agriculture, and Sea Ministry - partnerships with various stakeholder including institutions from public and private sector and civil society |  | -intra-ministerial group and structures from pre-implementation stayed ( - 1 year monthly meetings to identify priority areas) - EIPAS working group: monitoring of implementation progress and for delivering biannual reports to the Portuguese Government. All the actions implemented under the EIPAS scope should include an impact assessment. | - No formal evaluation  - preliminary re-salts of the impact of this strategy suggest reduction of almost 50% in the consumption of sweetened beverages with ≥8 g of sugar per 100 mL  - reduction contributed to decrease of 15% in total sugar consumption from these food products (more than 5600tons of sugar) |  |
| 2 | Throw et al. | 2011 | Fiji | national | SSB | case study, stakeholder interviews and policy document analysis | policy cycle, advocacy coalition framework for stakeholder analyses | 2006 | before informal discussions between Ministry of Health and Finance regarding possibility of soft drink tax in the context of a multi-sectoral national Non-Communicable Disease committee | 2007 Reduction of domestic excise tax due to industry lobbying; replaced by 3% fiscal import duty on raw material | revenues | external pressure (trade liberalisation) | import excise duty (5%) and excise duty (on locally manufactured products) of 5 c/l |  | taxation part of a new revenue initiative | no | state | population |  | x |  | Fiji Island Revenues and Custom Authority (FIRA) | top-down | FIRCA |  |  | FIRCA collected taxes along with other excise duties on tobacco and alcohol (linkage) | no information available due to lack of pricing and production data |  |
|  |  |  | Samoa | national | SSB | case study, stakeholder interviews and policy document analysis | policy cycle, advocacy coalition framework for stakeholder analyses | excise tax: 1984 along with tax for alcohol and tobacco (all other excise taxes were removed) -> 1984: 20%; 1998- 0.30 Tala/l; 2008-0.40 Talar/l, | two laws combined in 2007; both increased in 2008 due to budget deficit |  | revenues | budget deficit | excise tax (domestic production) and import tax duty |  | only 1 tax/ later both taxes combined | no | state | population | Ministry of Health raised awareness of importance of healthy eating before tax increase | x |  | Ministry of Finance increased the tax in line with Ministry of Trade advocacy for the use of excise taxes and GTS (good and service taxes) | top-down |  |  |  | Ministry of Revenue collects both taxes, tax linked to alcohol/tobacco -> no new structures | 2003-2007 - excise tax: ca US$ 3.5 mio; 2007 import excise: ca. US$ 170000; manufactures/importers -> passing on tax to consumer;  survey data: soft drink servings m/w per week from around 2.5 to just over 2 per week 1991-2003 | indirect effect: bottled water now cheaper (limited access to safe water -> bottled water appropriate alternative) |
|  |  |  | Nauru | national | SSB |  | policy cycle, advocacy coalition framework for stakeholder analyses | 2007 |  |  | discourage excessive consumption of sugar (and increase revenues) | health in combination with increase of budget | sugar levy of 30% | imported sugar, confectionery, carbonated soft drinks, cordials, flavoured milks, drink mixes | in combination with lift on levy on bottled water (to offset impact of tax on household budgets) | no | state | population |  | x |  | Ministry of Health proposed tax (due to high rates of diabetes and other chronic diseases) | top-down |  |  |  | tax mechanism already applied to alcohol, tobacco and petrol; collected at port with other import duties | tax was not passed on fully to customers -> cheap Asian products; price of bottled water still more expensive; locally produced drinking water not significantly cheaper (delivered by tanker trucks) |  |
|  |  |  | French Polynesia | national | SSB |  |  | 2002 | excise tax since 2006 -> redirected to general government budget with 80% earmarked for Ministry of Health budgeted (new elected governmental decision -> wanted most of tax revenue in general budget but EPAP had a cushion of unspent funds that had to be spent) |  | Funding for establishment pour la prevention (EPAP) (prevention fund) and to fund hospitals | health | excise tax, import tax | excise tax: sweetened drinks, beer  import tax: sweetened drinks, beer, confectionery, ice cream | range of taxes - on tax on soft drinks | no | state | population |  | x |  | support of wide range of ministries due to wide range of activities of the fund: public health, education, youth and culture, sport, family, road safety -> benefit 7 out of 17 ministries | top-down |  |  |  | collected through existing import-export mechanism | excise tax: US$ 10 mio, import tax US$ 4.2 mio/year |  |
| 3 | Falbe et al | 2020 | Berkeley, CA, USA | regional | SSB | Semi-structured interviews |  | 2014-> enforcement distribution: January effective but not enforced, March 2015 enforced, self-distribution January 2016 |  |  |  | health and revenue generation | excise tax of $0.01 per ounce of SSB distribution |  | tax | no | country level | population | communication strategy and outreach to companies affected | x |  | City government and SSBPPE | top-down but with societal support: City government decision that revenues should go to general fund to keep vote threshold at simple majority; but establishment of a SSBPPE to advise the city funding programs to further reduce SSB consumption and its consequences |  | -support of diverse societal stakeholders (parents, teachers, health professionals, Latinos others)  -early leadership engagement across multiple city departments (Finance, Attorney's Office, Public Health, City Manager's Office) |  | -simple tax calculation - hiring of a tax administration company to coordinate tax outreach and collection (cost 20% of tax proceeds) - after implementation start: part--time/later full-time program specialist in Berkeley Public Health Division - implementation plan inclusive communication strategy | within 1 year consumption decline in lower-income neighbourhoods; purchasing drop of 10% in supermarket | barriers mentioned: product definition: syrup and misperceptions about the use of the term "natural" sweeteners |

SSBPPE: SSB product Panel of Experts
