# Supplementary material for: Sugar-sweetened beverage tax implementation processes: results of a scoping review
Source: Health Res Policy Syst. 2022 Mar 24;20:33. doi: 10.1186/s12961-022-00832-3 (PMC8944035; doi:10.1186/s12961-022-00832-3)
Supplement: Supplementary file 4 — Additional file 4. Example of a search strategy for Medline. [file 12961_2022_832_MOESM4_ESM.docx]

# Appendix 4

**Database:** MEDLINE(R) and Epub Ahead of Print, In-Process & Other Non-Indexed Citations, Daily and Versions(R) **Date range:** 1946 to February 5, 2020 **Platform:** Ovid **Search date:** February 6, 2020

| **Search line** | **Query** | **Results** |
| --- | --- | --- |
|  |  |  |
| 1 | ("public polic*" or law* or "action program*" or regulation* or guideline* or "action plan*").ti,ab. | 1.309.626 |
| 2 | ((nation* or state* or government*) adj5 (policy or policies or policymak* or "policy mak*" or strateg* or recommendation*)).ti,ab. | 45.282 |
| 3 | exp public policy/ | 137.412 |
| 4 | exp policy making/ | 25.295 |
| 5 | or/1-4 | 1.468.739 |
| 6 | (implement* or enforc* or adapt* or application* or apply or applies or "policy formulation*" or "policy formation*" or "policy change*" or scale-up).ti,ab. | 2.152.253 |
| 7 | 5 and 6 | 193.033 |
| 8 | ((sugar* or "sugar-sweeten*" or "sugar sweeten*" or sweeten* or carbonated or fizzy or fruit) adj5 (drink* or beverage* or refreshment*)).ti,ab. | 7.547 |
| 9 | (SSB or lemonade* or coke or cola* or "energy drink*" or "soft drink*" or "sport* drink*" or "soda pop" or "flavo?r* water").ti,ab. | 16.265 |
| 10 | exp carbonated beverages/ | 2.873 |
| 11 | exp energy drinks/ | 708 |
| 12 | or/8-11 | 22.503 |
| 13 | 7 and 12 | 352 |

**Overview number of hits per database**

| **Database** | **Original search, number of hits** | **Deduplicated search, number of hits** |  |
| --- | --- | --- | --- |
| Medline | 352 | 352 |  |
| EMBASE | 206 | 73 |  |
| PsycInfo | 91 | 46 |  |
| CINAHL | 228 | 72 |  |
| EconLit | 28 | 14 |  |
| ASSIA | 49 | 25 |  |
| ERIC | 640 | 6 |  |
| PAIS | 68 | 38 |  |
| WoS | 311 | 27 |  |
| Scopus | 409 | 356 |  |
|  |  |  |  |
|  | **2.382** | **1.009** | **total** |
|  |  | 1.373 | discards |
